# Antimicrobial stewardship challenges at a referral Hospital in Western Kenya: insights from a Kakamega–Cambridge health partnership

**DOI:** 10.3389/fpubh.2025.1731706

**Published:** 2026-01-20

**Authors:** Anthony Sifuna, Christine Wanjala, Nicholas Nyabera Mogoi, Lindsey Olima, Rose Malaba, Evelyn Brealey, Lucy Mandillah, Frida Njeru, Sarah Kindiki, Robin Omedo, Roselyne Abwalaba, Cristiano Serra, Bobbak Rabiei, Bonface Nyumbile, Peter Nyongesa, Martin Welch

**Affiliations:** 1 Department of Medical Biochemistry, Masinde Muliro University of Science and Technology, Kakamega, Kenya; 2 Kakamega County General Teaching and Referral Hospital, Kakamega, Kenya; 3 Cambridge Global Health Partnerships, Addenbrooke’s Hospital, Cambridge, United Kingdom; 4 Royal Papworth Hospital NHS Foundation Trust, Cambridge, United Kingdom; 5 Department of Biochemistry, University of Cambridge Department of Medicine, Cambridge, United Kingdom

**Keywords:** antimicrobial resistance, antimicrobial stewardship, antimicrobial use, health partnerships, point prevalence survey

## Abstract

**Objective:**

To identify ways of improving antimicrobial stewardship at Kakamega County Teaching and Referral Hospital (KCTRH) in western Kenya.

**Methods:**

A knowledge and awareness test was conducted among surgical ward healthcare workers to assess their understanding of how to counter antimicrobial resistance. In parallel, the global Point Prevalence Survey (PPS) was used to understand antibiotic prescription practices at the referral facility. Patient records from the adult surgical and pediatric surgical wards were examined for information on antibiotic prescription and usage. A total of 47 healthcare workers and 106 patient records were examined.

**Results:**

Healthcare workers showed the highest competence when tested on the appropriate use of antimicrobials, with a mean score of 78.7%. However, their understanding of surveillance and monitoring was lower, with a mean score of 60.2%, followed by awareness and education (which achieved a mean score of 64.5%). The majority of prescriptions (62.3%) were for empiric treatment, with a lack of laboratory data to support targeted therapies. Prolonged antibiotic courses were noted for surgical prophylaxes with over 83% of patients being administered antibiotics for more than 1 day. The survey also revealed over-prescription of broad-spectrum agents such as ceftriaxone at 36.7%.

**Conclusion:**

There is a high use of antibiotics, but a weakness in AMR monitoring at KCTRH. Better use of laboratory testing (AMR profiles for infecting bacteria, etc) is recommended as the best way to improve patient management and deployment of antibiotics.

## 1 Introduction

Health systems in sub-Saharan Africa (SSA) face numerous challenges, including inadequate human resources, insufficient funding, poor infrastructure, and a high burden of disease (1–5). On the other hand, antimicrobial resistance has emerged as a global health concern that requires concerted effort to control or mitigate (6–8). Nevertheless, SSA continues to face weak healthcare systems, poverty and inadequate infrastructure, which have compounded the fight against antimicrobial resistance (9). However, for this fight to realize success, efforts need to be directed toward strengthening surveillance systems, promoting responsible antimicrobial use, improving infection prevention and control, and fostering collaboration across various sectors. It is with this vision that the Kakamega–Cambridge health partnership was established.

The Kakamega County Teaching and Referral Hospital (KCTRH), like many healthcare provision facilities in SSA, is faced with limited resources (both financial and human). KCTRH is a level-5 hospital with a 500 in-patient bed capacity and around 1,000 out-patients seen per day. As a referral center, most of the surgical procedures in Kakamega County are done at KCTRH. However, KCTRH does not have an established AMR surveillance program. Therefore, and although it has an established antimicrobial stewardship team, their efforts toward effective delivery at the healthcare facility remain limited. The absence of these data continues to undermine the fight against AMR and exacerbates the spread of hospital-acquired infections and antibiotic resistance. This, in turn, has a negative clinical and cost implication for patients, communities and the healthcare system (10, 11). To circumvent this challenge, staff at KCTRH teamed up with staff at the Cambridge University Hospitals NHS Foundation Trust (Cambridge, United Kingdom) to engage in an effort to improve and strengthen antimicrobial stewardship (AMS) at the facility. An AMS program aims at: (i) optimizing antibiotic use, (ii) promoting appropriate prescribing practices, (iii) improving patient outcomes, (iv) reducing healthcare costs, and (v) minimizing the development and spread of antimicrobial resistance (AMR) (12).

The team carried out surveys to assess the knowledge and awareness of healthcare staff at the facility regarding AMS and antibiotic use in the surgical wards. This study focused on the surgical wards, because in our settings, the surgical department is one of the heavy consumers of antibiotics (12). To qualify antibiotic use, the global Point Prevalence Survey (PPS) was used. The PPS is a study that measures the proportion of individuals in a population who have a specific characteristic such as a disease or condition at a single, specific point in time. It can be used to monitor healthcare-associated infections and antimicrobial use in hospital. Additionally, it can also show how health facilities comply with the AWaRe classification system, which categorizes antibiotics under three groups of Access, Watch and Reserve as part of AMS (13). The Access group contains antibiotics used in the first- and second-line treatment of infections. The Watch group contains broad –spectrum antibiotics with a higher potential of developing resistance. The Reserve group contains last-resort antibiotics used for multi-drug resistant infections.

This study reports on knowledge and awareness among healthcare workers of these five key strategic objectives of the Global action plan on AMR prevention, and on efforts made by the partnership to establish a program for monitoring and quantifying antimicrobial use and compliance on the surgical wards. The five strategic objective on AMR include: Objective 1: Improve awareness and understanding of AMR through effective communication, education and training. Objective 2: Strengthen the knowledge and evidence base through surveillance and research. Objective 3: Reduce the incidence of infection through effective sanitation, hygiene and infection prevention measures. Objective 4: Optimize the use of antimicrobial medicines in human and animal health. Objective 5: Develop the economic case for sustainable investment that takes account of the needs of all countries, and increase investment in new medicines, diagnostic tools, vaccines and other interventions (14).

This work contributes to the current global health agenda on antimicrobial resistance by demonstrating how international partnerships can bolster local health systems, especially those in lower and middle-income countries (LMICs) where resource constraints often frustrate the implementation of good AMS.

## 2 Materials and methods

### 2.1 Study site

The study was conducted at KCTRH; a regional referral hospital and also a teaching and research facility for the Masinde Muliro University of Science and Technology (MMUST) schools of Medicine, Public Health, Biomedical Sciences & Technology, and Nursing. Kakamega County has a population of 2 million people, with the majority residing in the rural areas. Kakamega County Teaching and Referral Hospital is the largest referral hospital in Western Kenya. The facility receives approximately 15,000 patients monthly and has a bed capacity of 500. The selection of the surgical ward was based on its high antibiotic use related to surgical prophylaxis and post-operative care. Previous data shows that in LMICs, surgical units are frequent contributors of inappropriate antibiotic use and hence key targets for AMS.

### 2.2 Study design

#### 2.2.1 Point prevalence survey

To understand antibiotic use at the surgical ward, a Point Prevalence Survey (PPS) was conducted on 22nd April 2022, using the Global Point Prevalence Survey (Global-PPS) standardized method (February 2022 version). This method was used to assess antimicrobial prescribing quality, healthcare-associated infections (HAIs), and antimicrobial resistance (AMR) see www.Global-PPS.com.

#### 2.2.2 Sampling

The survey was conducted across all in-patient beds in the surgical wards, specifically examining records from the adult surgical ward (ASW) and the pediatric surgical ward (PSW). The adult surgical wards comprised of the general, orthopedic and gynecology surgical wards; whereas pediatric surgical ward represented all case-mix. Acuity comprised of both emergency and elective case. Medical patient’s charts with incomplete or missing data were excluded from the study.

#### 2.2.3 Inclusion and exclusion criteria

##### 2.2.3.1 Inclusion criteria

All patients who were admitted to the hospital and present in the surgical wards at 8:00 a.m. on the survey day 22nd April 2022, having stayed overnight.

All patients receiving antimicrobial treatment or prophylaxis (surgical or medical) at the time of the survey.

##### 2.2.3.2 Exclusion criteria

Outpatients; in-patients discharged before 8:00 a.m. on the survey day; day-surgery patients.

#### 2.2.4 Data collection and quality control

Data was collected manually from the prescription charts and patient notes using a retrospective approach to gather surgical prophylaxis information from patient treatment reports. The data collection form is available at https://www.global-pps.com/documents/.

##### 2.2.4.1 Data collected

The following information was recorded: Patient demographics: gender and age; antimicrobial details: prescribed antimicrobials, and diagnosis; prescription-related quality indicators; start and stop date; guideline compliance. Patients with incomplete or missing data were excluded from the study.

##### 2.2.4.2 Quality control

The enumerators used in this study underwent a two-day training on the PPS methodology at KCTRH prior to data collection to ensure standardized procedure. Data was collected by these trained staff and then entered into the Global PPS web-based application for processing. This study was part of a collaboration between KCTRH and Cambridge NHS Foundation Trust, facilitating capacity building and collaborative interpretation of research data.

#### 2.2.5 Data analysis

Data was presented as proportions of each group. The Chi-square test was used to determine associations between variables.

#### 2.2.6 Knowledge and awareness survey

A knowledge and awareness survey was conducted on 9th March 2022, to assess staff understanding of AMS. The survey was conducted by collecting data using a self-administered, pretested questionnaire based on a dichotomous response format (True/False; Supplementary data S2). Fifteen (15) survey questions were developed primarily from WHO online materials: the AMS competency frameworks and the AMS programs in health-care facilities in low- and middle-income countries - a practical toolkit. The questions were structured around five key AMS strategic objectives: (i) governance and coordination; (ii) awareness and education; (iii) infection prevention and control (IPC); (iv) appropriate use of antimicrobials; (v) surveillance and monitoring. The questionnaire was reviewed by experts from both partner institutions (KCTRH and Cambridge NHS Foundation Trust) to ensure content validity. The questionnaire was piloted at the pharmacy department to test for question clarity and ease of administration.

##### 2.2.6.1 Participants and data collection

The survey targeted staff who were on duty in the wards on the day of the assessment selected from the respective duty roster of the day by balloting. A total of 50 participants in the survey. The questionnaire was self-administered by the participating staff. Nevertheless, participation was voluntary, non-participation had no consequences, and that responses were treated confidentially. The questionnaires were numbered for internal reference, allowing them to be traced back to the respondents and their professional categories (e.g., Doctors, Nurses, and Pharmacists etc.). The questionnaire number and named individuals is only accessible by the research team for future follow-up assessments. There were no incentives provided to the participants.

##### 2.2.6.2 Data analysis

All questionnaires were scored based on the correct/incorrect responses. Analysis was conducted based on the participants’ professions and categorized according to the five key AMS strategic objectives. Data were exported and analyzed using SPSS (Version 30). One-way ANOVA was applied to compare data between groups, specifically. A *p*-value of <0.05 was considered statistically significant. Where appropriate, proportions and variance values were reported with 95% confidence intervals.

### 2.3 Ethical considerations

This study was approved by the Kakamega County Research and Ethics Committee as a quality improvement activity. Nevertheless, human data were collected and analyzed, albeit under a QI framework with a consent waiver.

## 3 Results

### 3.1 Knowledge and awareness among healthcare workers

In this study, a total of 50 healthcare workers were invited to participate in the assessment of knowledge and awareness. The questionnaire is shown in SI data S2. Out of these, a total of 47 healthcare workers participated in the knowledge and awareness test. Three of the healthcare workers that did not respond were from the adult orthopedic surgical ward. The professionals that participated in the study and their role descriptions are presented in Table 1A. The majority (59.6%) of the healthcare workers registered 5–10 correct answers, out of the 15 questions that were presented to them, nevertheless, 40.4% registered 11–15 correct answers. Among the professions, doctors had the highest mean score of correct answers at 11.5, although this cadre recorded the highest variation in scores with a standard deviation of 2.17. Pharmaceutical technologists and students, registered a mean score of 9.7, however the students recorded a higher standard deviation of 1.58, against 0.58 for pharmaceutical technologists as shown in Table 1B. There was however no significant difference in performance among the cadres (*p* = 0.29). The AMS strategic objective appropriate use of antimicrobials recorded the highest mean performance rates among healthcare workers at 78.7%, with whereas surveillance and monitoring registered the lowest at 60. 25% as shown in Table 1C.

**Table 1:** Mean score for different healthcare workers in the surgical wards.

Healthcare professionals by cadre	N	
A. Participation and scores by Cadre		
Doctor	6	
Nurse	10	
Pharmacist	8	
Pharmaceutical technologist	3	
Clinical officer	4	
Student	9	
Laboratory staff	5	
Interns doctors	2	
Overall performance		
Scores	Counts	%
11 to 15	19	40.4
5 to 10	28	59.6
0 to 4	0	0
Total	47	

	Mean	Variance	*p* value
B. Mean scores against performance per profession			
Doctor	11.5	4.7	*p* = 0.29
Nurse	10.1	1.9	
Pharmacist	11.4	3.4	
Pharmaceutical Technologist	9.7	0.3	
Clinician	11.0	2.0	
Student	9.7	2.5	
Laboratory Staff	10.0	4.5	
Interns	10.0	0.0	

Strategic objectives	Number of questions	Mean (%)	*p* value
C. Mean scores representing performance per AMS strategic objective			
Governance & coordination	4	72.9	*p = 0.86*
Awareness & education	3	64.5	
Infection prevention & control	3	73.0	
Appropriate use of antimicrobials	1	78.7	
Surveillance & monitoring	4	60.25	

A clinician is trained middle level healthcare workers (not a medical doctor) trained to take patient histories, conduct physical examinations, diagnose common ailments, and prescribe appropriate treatment. Intern is a career development position at which they acquire hands-on experience, networking opportunities, and a chance to explore different industries (in this only intern doctor’s tool part). This table shows the total number of healthcare workers who participated in the knowledge and awareness test and their respective scores. One-way ANOVA was used to compare mean scores between professional scores (*p* < 0.05). There was no statistically significant difference in performance among the health professionals assessed.

### 3.2 Overall antimicrobial use

A total of 106 patient records (99 adult ward and 7 pediatric ward) were examined. Of these, 53 patients were being treated with at least one antimicrobial on the day of the survey, with about 47.2% of the patients being prescribed antimicrobials for systemic use, and 3.8% using nitroimidazole derivatives. Overall, antibiotic usage at the KCTRH surgical wards was 48.5 and 28.6% for adult and pediatric surgical wards, respectively. There were 98 antibiotic prescriptions on the day of the survey; the most widely prescribed class of antibiotics were *β* − lactams (69%), whereas quinolones were the least prescribed (1%; Table 2).

**Table 2:** Overall antimicrobial use KCTRH surgical wards.

Drug	Frequency of prescription (%); *N* = 98
β − lactams	69
Macrolides, lincosamides and streptogramins	6
Aminoglycosides	3
Quinolones	1
Other antibacterials	21

This table shows the overall antmcirobial use among the total patients. The most widely prescribed class of antibiotics were β − lactams and quinolones the least prescribed.

### 3.3 Individual antibiotic usage patterns

Third-generation cephalosporins were the most prescribed class of antibiotics at the time of the survey, followed by β lactamase-resistant penicillins and imidazole derivatives. Macrolides and fluoroquinolones were the least prescribed (Table 3). Among the cephalosporins, ceftriaxone represented the highest usage (81.8%). Interventions for which antibiotics were deployed in the adult surgical ward (ASW) included healthcare-associated infections (HAIs) and surgical prophylaxis (SP). When comparison was made using Chi square, there was an association (*p* = 0.000) between antibiotic sub-group used and the intervention (HAI and or SP).

**Table 3:** Proportion (%) of total antibiotic use and reason for antibiotic intervention.

Antibiotic sub-groups (*N* = 98)	OP	HAIs	SP
Penicillins with extended spectrum	3.1	12.5	-
β lactamase-resistant penicillins	20.4	4.2	25
First-generation cephalosporins	7.1	-	10.3
Third-generation cephalosporins	37.8	54.2	32.4
Macrolides	1	4.2	-
Lincosamides	5.1	-	7.4
Other aminoglycosides	3.1	-	1.5
Fluoroquinolones	1	-	1.5
Tetracyclines	-	-	-
Imidazole derivatives	21.4	25	19.1

OP, Overall proportion; ASW, Adult surgical ward; HAIs, Antibiotic prescribed to address healthcare-associated infection; SP, Antibiotic prescribed for surgical prophylaxis. This table shows the most prescribed class of antibiotics at the time of the survey. Third generation cephalosporins were the most prescribed followed by β-lactamase resistant penicillins and imidazole derivatives. Macrolides and fluoroquinolones were the least prescribed.

### 3.4 Use of antibiotics in surgical interventions

**Figure 1:**
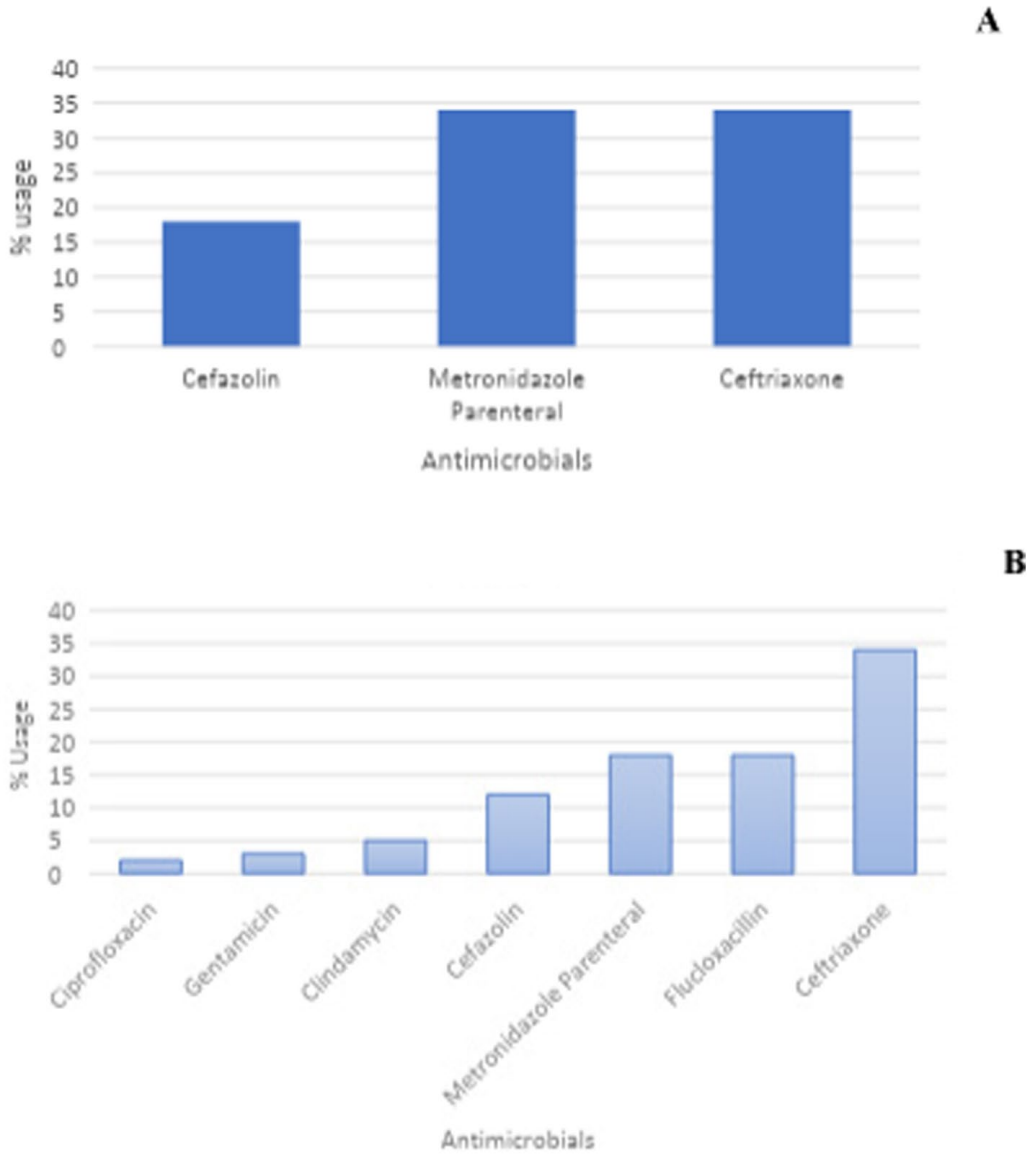
Prophylactic antibiotics prescribed for surgical interventions. **(A)** Genito-uinary male surgery; **(B)** surgery.

Ceftriaxone was the most frequently prescribed prophylactic antibiotic for surgical interventions at 34%. Flucloxacillin and metronidazole parenteral were second, at 18%. Cefixime, ciprofloxacin and erythromycin were the least prescribed as shown in Figures 1A,B. Metronidazole parenteral registered high usage for genito-urinay male surgical interactions at 34%.

Out of the 53 patients who were prescribed antibiotics, only 20 (37.7%) had a defined diagnosis before prescription; all other prescriptions were for empirical antibiotic therapy. Obstetric/gynecological related infections were the most common for which antibiotics were prescribed following some form of diagnosis, representing 85% of all prescriptions accompanied by diagnosis. Most of the diagnosis was based on hemogram indictors. All community-acquired infections (CAIs) and hospital-acquired infections (HAIs) surveyed were treated empirically, as shown in Table 4.

**Table 4:** Infections treated following diagnosis **(A)** and empirical antimicrobial use for community- and healthcare-associated infections by type of treatment **(B)**.

Diagnosis	N	%	Overall % (53)
A. Diagnosis done			
Obstetric/gynecological infections	17	85	32.1
Infection of central nervous system	1	5.0	1.8
Genitourinary tract male infections	1	5.0	1.8
Intra−abdominal sepsis	1	5.0	1.8
B. Empiric prescription			
Type	N	%	Overall % (53)
Community-acquired infections	6	100	11.3
Healthcare-associated infections	27	100	50.9

### 3.5 Duration of surgical antibiotic prophylaxis administration in adults and children

The majority of the prescriptions were for therapies of more than 1 day, namely; 100% for central nervous system infection prophylaxis; 83% for surgical prophylaxis and 81% for plastic and orthopedic surgery prophylaxis (Table 5). However, when compared using Chi square, there was no statistical significant association between medical condition and duration of antibiotic prophylaxis (*p = 0.97*).

**Table 5:** Duration of surgical antibiotic prophylaxis in adults and children (% of prescriptions).

Medical Condition	Single dose	1 Day	>1 day
Surgical Prophylaxis (*N* = 30)	14	3	83
Genito-urinary prophylaxis (*N* = 4)	33	-	67
Prophylaxis for plastic and orthopaedic surgery	14	5	81
CNS prophylaxis	-	-	100

CNS, infection of central nervous system. Table showing duration of surgical antibiotic prophylaxis administration in adults and children.

### 3.6 Overall top 5 antibiotics used (ATC J01), according to WHO AWaRe classification

The study showed that 60% of the antibiotics prescribed were in the Access category, whereas 40% were in the Watch category. Ceftriaxone in the under Watch category was the most used antibiotic in surgical wards, at 36.7%, followed by metronidazole and flucloxacillin at 21.4 and 20.4%, respectively (both of these antibiotics are in the Access category of the WHO AWaRe classification) (13). Cefixime, ciprofloxacin and erythromycin were the least prescribed (<1%). These antibiotics are all on the Watch WHO AWaRe classification (Supplementary data S1). No prescriptions were recorded for antibiotics on the Reserve category (Table 6).

**Table 6:** Overall top 5 antibiotics used (ATC J01), according to WHO AWaRe classification (13).

Antibiotic	WHO AWaRe classification	% Use
Metronidazole	Access	21.4
Flucloxacillin	Access	20.4
Cefazolin	Access	7.1
Clindamycin	Access	5.1
Amoxicillin	Access	3.1
Ceftriaxone	Watch	36.7
Cefixime	Watch	1
Ciprofloxacin	Watch	1
Erythromycin	Watch	1

Table showing % use of antibiotics with respect to the WHO AWaRe classification.

### 3.7 Antibiotic quality indicators

Out of 98 prescriptions, 87 included notes indicating the reason for the prescription, but only 20 (40.8%) were compliant to the WHO guidelines (15). Fifty two of the prescriptions (54.7%) showed the stop or review dates, as shown in Table 7.

**Table 7:** Antibiotic quality indicators.

Indicator	N	%
Reason documents in notes	87	91.6
Guidelines’ Compliant	20	40.8
Stop/reviews date document	52	54.7

This table shows indicators for antibiotic quality, including reason for prescription, in line with WHO compliance.

## 4 Discussion

The main goal of this partnership was to quantify the antimicrobial use at KCTRH surgical wards, a challenge that many health facilities in LMICs have yet to accomplish. Our findings suggest slightly higher overall antibiotic use at KCTRH (48.7%), compared with Kenyatta National Hospital (KNH; 43% antibiotic usage) and Moi Teaching and Referral Hospital (MTRH; 47% antibiotic usage) but lower antibiotic usage than Coast Provincial General Hospital (CPGH; 52%) (16). These differences may be attributable to the levels of care at each facility and patterns of referral. Variation in antibiotic overuse across different types of healthcare settings is a global phenomenon, observed not only in the specific settings of the study but also in developed countries (17, 18). These persistent variations in antibiotic overuse can be rooted in several underlying systemic and social issues (17)^,^ including: structural risks and perverse incentives, social norms, missing infrastructure and patient poverty. Additionally, variation in antibiotic usage can be attributed to several factors, largely revolving around the prescriber and the patient namely; physician experience and individual practice patterns, time constraints, physician perceptions and attitudes, colleagues’ influence, and patient-related factors (perception and attitudes toward antibiotics) (18). In this study, findings point to an increased use of antibiotics as the healthcare facility level (based on Kenyan classification) is descended, with tertiary facilities being at the peak of the health facility classification pyramid in Kenya. Tertiary hospitals (Level 6) typically operate with larger budgets due to their national scope and highly specialized services. Their funding is primarily sourced from national government transfers, whereas County hospitals (level 5) receive a specific allocation from their respective county budgets (19). Significantly, Level 6 facilities enjoy greater autonomy in deploying their allocated resources than Level 5 hospitals, potentially enabling them to dedicate more funding to antimicrobial stewardship programs.

Importantly, this study shows the value of transnational collaboration in addressing the challenge of AMR. Crucially, in the Kakamega-Cambridge partnership, shared methodologies have been used to change practice and build capacity in the area of antimicrobial stewardship. Interestingly, our survey suggested above average knowledge about appropriate use of antimicrobials among healthcare workers at KCTRH, but lower levels of awareness about training in antimicrobial resistance and stewardship, or about the importance of surveillance and monitoring. These may be possible factors driving high antimicrobial usage at the facility. These challenges are further compounded by the limited information available to clinicians to guide the treatment and management of patients. In Kenya, public health facilities below the tertiary hospitals are poorly resourced, which again, could influence the healthcare services provided at these levels. For instance, clinical microbiology laboratories are poorly supported, particularly in terms of human resources, as well as diagnostic equipment (20). Irrational antibiotic use presents a complex problem globally, affecting not only LMICs but also developed nations, necessitating a multifaceted approach (21, 22). A promising approach for optimizing antibiotic use is improving education for healthcare professionals (23, 24). This improvement could include enhancing medical training curricula in both medicine and pharmacy. Specifically, it is becoming important to incorporate education on pharmaceutical promotion, including: strategies used by companies; the impact of these strategies on prescribing and dispensing; and practicing evidence-based medicine (25)^.^ Unfortunately, these emerging educational issues have not been sufficiently emphasized in Kenya. Addressing them is becoming increasingly vital for stakeholders in the health sector, particularly within the context of partnerships like the Kakemega–Cambridge collaboration. Additionally, training of healthcare personnel in specific courses, workshops and events designed explicitly for healthcare workers (HCWs) involved in managing patients (such as surgeons, general practitioners) is becoming more critical (26, 27). The Kakemega–Cambridge collaboration will therefore aim to develop such programs to strengthen AMS compliance at KCTRH.

Multifactoral approaches have nevertheless been suggested to maximize implementation of interventional efforts targeting HCW. Developing intrahospital protocols and guidelines have been shown to promote strong adherence to programs such as influenza vaccination among splenectomy patients of over the years (28). This partnership therefore will aim to develop guidelines and intrahospital protocols to strengthen antimicrobial stewardship programs including prudent antimicrobial use. Currently the absence of hospital antibiograms and infections prevention and control (IPC) policy continues to limit clinicians choices on the most effective optimal empirical treatment regimens (29), and prevention of healthcare-associated infections (30). Implementing such programs in healthcare settings in Kakamega County can reduce the rise of antimicrobial resistance, minimize healthcare costs and ensure a safe and high-quality care environment.

In our study, only 37% of patients had a recorded diagnosis, and most of these diagnoses were based on hematology. Some of the hematological parameters monitored include changes in white blood cell count (leukocytosis or leukopenia), specific immune cell ratios like the neutrophil-to-lymphocyte ratio (NLR) and platelet-to-lymphocyte ratio (PLR), and changes in hemoglobin and platelet levels. Nevertheless, these tests are dependent on availability of laboratory reagents, and therefore not regularly reported for all patients. This limits diagnostic capacity, which subsequently impacts on the ability to provide targeted therapy. Consequently, even though knowledge on antimicrobial use scored highly, it remains the case that healthcare workers have limited information on which to make informed decisions regarding antibiotic use, but relied on the antibiotic prescription algorithms provided by the hospital to guide the empirical antibiotic prescription (31)^.^

In this survey, all (100%) of the antibiotic treatments targeting hospital-acquired infections (HAIs) and community-acquired infection (CAIs) were based on empirical decisions. These high rates of empirical antibiotic usage at the healthcare facility serves to bring to the fore weaknesses in implementation of institutional or national antibiotic usage guidelines at secondary healthcare facilities in Kenya. Empirical prescription for both CAIs and HAIs underscores the lack of microbiology-based services, which limits the implementation of antibiotic usage guidelines (32, 33). This challenge may have been made worse in the wake of COVID -19, with mean rate of antibiotic use in COVID-19 management is 74.0% and only 17.6% of patients had secondary infections (34). Overall, proper management strategies need to be established by healthcare system institutions and policymakers, to sustain requirements for healthcare delivery and mitigate persistence misuse of antibiotics in LMIC healthcare institutions. This is urgent as witnessed during the COVID -19 pandemic, healthcare demand will probably overload the insufficient capacity of the public health service, previously weakened by resource limitations and pandemic bursts (35). Therefore, implementing measures to track the use of antibiotics and comply with the WHO’s guideline to promote antibiotic stewardship are critical strategies for combatting antimicrobial resistance and bolstering pandemic preparedness.

The use of third generation cephalosporins (3GC) specifically ceftriaxone recorded in this study is commensurate with other studies conducted in referral hospitals in Kenya and the region (16, 17, 33, 36) and is a major concern in healthcare systems in SSA. This is because ceftriaxone is not indicated for surgical prophylaxis (37). However, in SSA, poor infection prevention and control practices have been attributed to result in extensive inappropriate use of 3GC to increase clinicians’ confidence while conducting surgical operations (33, 38–40). This practice may contribute to the increased resistance to 3GCs among microbes in hospitals and the environment in the region (41–44). Resistance to 3GCs undermines treatment and exacerbates poor outcomes in SSAs with limited alternative therapies, and calling for immediate interventions including enhanced surveillance, improve IPC practices and strengthen AMS programs (45, 46).

Our findings show that the majority of antibiotic prescriptions for surgical prophylaxis were for more than 1 day of treatment. This appears to be common practice in Kenya, as also shown in previous studies (32, 47, 48). This is considered to be high, as per WHO recommendations, which recommends a single dose to be administered 1 to 2 h before surgery (37). Prolonged use of antibiotics for surgical prophylaxis may lead to unfavorable drug reactions and further enhance selection of antibiotic resistance. KCTRH has an established Medicines and Therapeutics Committee (MTC) responsible for guiding and approving formularies in the hospital. The choice of surgical prophylactic prescriptions documented in this study presents a picture of the challenges faced by such committees in poorly- resourced settings. The high levels of ceftriaxone usage may also be linked to behavioral factors, since clinicians may prefer to use antibiotics with a broader spectrum of action to ensure patients are covered for both Gram-positive and Gram-negative bacteria.

## 5 Conclusion

This study revealed a high prevalence of antibiotic usage at KCTRH, and highlights several areas where improvement is needed. Key emerging issues are that the majority of prescriptions remain empiric and do not follow the WHO recommended targets. Additionally, we note the prolonged duration of antibiotic use in surgical prophylaxis, as well as over prescription of broad spectrum agents such as ceftriaxone. These issues appear linked with the resource limitation of the facility, which has limited access to surveillance and monitoring information. Our study also emphasizes the need for ongoing awareness and educational support regarding AMS.

Our data are commensurate with the notion that inadequate resourcing in the LMIC healthcare setting is associated with greater levels of empirical antibiotic prescription, low microbiological diagnostics and poor enforcement of standard treatment guidelines. Our study also provides actionable data for policy makers, and shows that there is a need for greater public awareness and education to promote improved antibiotic stewardship, especially among healthcare workers. In addition to its international collaborative nature, another key strength of this collaboration lies in the deployment of validated tools such as the PPS. However, the study also has limitations, which we hope to address in future work. These limitations include its cross-sectional design and the lack of accompanying microbiological diagnostic data that can be used in the confirmation of treatment indications. Additionally, our findings primarily apply to surgical wards and should not be extrapolated to the entire hospital without caution.

### 5.1 Recommendations

To enhance good antimicrobial stewardship and minimize the spread of AMR, we recommend (i) establishment of a functional AMR surveillance program at the KCTRH to strengthen surveillance and monitoring, (ii) access to awareness and educational support regarding AMS and the threat of AMR, (iii) improved infection prevention and control mechanisms, (iv) prudent prescription of antibiotics and laboratory testing, (v) develop and implement a hospital AMR action plan.

## Data Availability

The original contributions presented in the study are included in the article/supplementary material, further inquiries can be directed to the corresponding author.
